# The Piezo1-Ca2+-PI3k/Akt signaling axis as a context-dependent mechanotransduction node during skin wound healing

**DOI:** 10.1016/j.isci.2026.115598

**Published:** 2026-04-07

**Authors:** Shangqing Wang, Yahui Li, Yaxi Han, Shuai Li, Ruonan Zheng, Yao Jia

**Affiliations:** 1 Department of Plastic Surgery, The First Hospital of Shanxi Medical University, Taiyuan, Shanxi, China; 2 First Clinical Medical College, Shanxi Medical University, Taiyuan, China

**Keywords:** mechanobiology, molecular biology, bioengineering

## Abstract

Mechanical forces are fundamental drivers of cutaneous tissue repair; however, the molecular mechanisms translating physical cues into fibroblast responses remain incompletely understood. This review examines the Piezo1-PI3K/Akt signaling axis as a putative convergent context-dependent mechanotransduction node, through which multiple mechanical inputs may be transduced into intracellular biochemical cascades. Direct evidence in dermal fibroblasts is strongest for the integrin-FAK arm; we synthesize findings across cell types to propose a multi-pathway framework encompassing four molecular bridges: CaM/CaMK-mediated activation, a synergistic integrin-FAK loop, calpain-mediated regulation, and a Panx1-ATP-P2Y2 paracrine pathway. Beyond signal relay, this axis may function as a spatiotemporal encoder coordinating fibroblast proliferation, migration, metabolic reprogramming, and myofibroblast differentiation. By contrasting physiological healing with dysregulated mechanotransduction in chronic wounds and hypertrophic scars, we highlight the therapeutic relevance of this axis and propose that its modulation may offer a strategy to restore mechanical homeostasis and improve clinical outcomes.

## Graphical abstract

**Figure:**
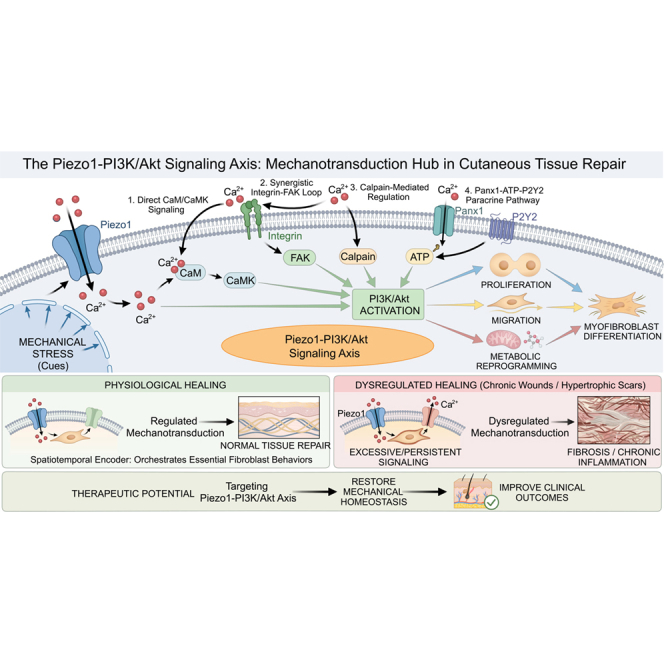

## Introduction

### Clinical background and challenges

The skin plays a vital role in preserving the homeostasis of an organism, making its structural integrity essential. When tissue is damaged, the body usually triggers a well-orchestrated series of biological reactions aimed at facilitating repair. Nevertheless, certain pathological conditions like diabetes, aging, and extended periods of immobility can greatly hinder this healing process, leading to wounds that remain stuck in the inflammatory stage and evolve into chronic non-healing ulcers.^1^ These wounds not only resist closure but are also highly vulnerable to ongoing infections. Examples of such persistent wounds include diabetic foot ulcers, pressure sores, and chronic venous ulcers, which not only drastically affect the quality of life for patients but also pose a significant risk of amputation, creating a considerable economic strain on healthcare systems worldwide.^2^

Present-day clinical treatment methods, although offering some relief, are increasingly showing their shortcomings.^3^ Standard practices like debridement and wound dressing often require lengthy treatment periods with less than ideal results; traditional antibiotic treatments can control infections but are significantly hindered by the rise of bacterial resistance. Additionally, supportive techniques such as negative pressure wound therapy, which enhance the local mechanical environment and boost microcirculation and metabolic support, have proven effective in certain instances.^4^ However, their high costs and complex procedures restrict broader use.^5^^,^^6^ As a result, research is now shifting from a focus solely on biochemical signaling to exploring the convergence of biophysical and biochemical pathways.

### The role of mechanical forces in cutaneous wound healing

Recent research has highlighted the essential role of biophysical signals in the process of skin repair.^7^ Among these signals, mechanical force has been shown to influence the regeneration of skin tissue through pathways known as mechanotransduction. Available evidence suggests that this force may intricately modulate the activities of fibroblasts, keratinocytes, and vascular endothelial cells, which in turn facilitates the remodeling of the extracellular matrix (ECM) and promotes tissue healing.^8^ Additionally, the careful management of mechanical stress appears to be crucial for maintaining a balance in matrix deposition, contributing to the transformation of fibroblasts into myofibroblasts, and potentially preventing issues such as pathological fibrosis and hypertrophic scarring.^9^^,^^10^ Nevertheless, the mechanisms by which mechanical forces are converted into intracellular biochemical signals that direct the healing process remain a significant area of inquiry that requires further investigation.

### Piezo1: The transducer of mechanical force to biochemical signals

In 2010, researchers discovered Piezo proteins, specifically Piezo1 and Piezo2, as part of a new category of mechanically activated cation channels (MACs).^11^ Distinct from traditional voltage-gated or ligand-gated channels, Piezo1 features a unique homotrimeric structure.^12^ When activated, Piezo1 allows cations, mainly Ca^2+^, along with Na^+^, K^+^, and Mg^2+^, to enter the cell.^11^ As a common second messenger, Ca^2+^ initiates various signaling pathways by altering its concentration over time and space, influencing critical pathways such as calcineurin/NFAT, YAP/TAZ, and PI3K/Akt.^13^^,^^14^ Research has suggested that the activation of the PI3K/Akt pathway may be linked to the proliferation, movement, and survival of fibroblasts,^15^ although the precise mechanisms in dermal fibroblasts remain to be fully characterized. Additionally, multiple studies have shown that the activation of Piezo1 can enhance Akt phosphorylation in various cell types, indicating a functional relationship between the two.

However, the following key questions remain to be systematically elucidated:

What are the molecular mechanisms that connect Piezo1-mediated Ca^2+^ signaling to the PI3K/Akt pathway? How does this interaction occur at the molecular level?

How does this signaling pathway collectively manage various functional responses of fibroblasts, including their growth, movement, transformation, and metabolic adjustments?

Is this signaling pathway disrupted in persistent non-healing wounds or abnormal scars? Could it be a potential target for targeted treatment?

Given these factors, this analysis seeks to thoroughly clarify the possible molecular pathways involving the Piezo1-Ca^2+^-PI3K/Akt signaling pathway in skin fibroblasts and its role in the process of wound healing. By conducting a comprehensive review, we aim to offer fresh insights into the mechanobiological processes underlying skin wound healing and lay the groundwork for innovative treatment approaches that focus on regulating mechanotransduction.

## Structural basis and mechanogating mechanisms of Piezo1

### Physical basis of mechanotransduction

Mechanotransduction is a complex mechanism that transforms physical mechanical forces into biochemical signals within cells, playing a vital role in maintaining physiological balance in mammals. This intricate regulatory system is both vast and deep.^16^ The process depends on how the cell membrane reacts to mechanical forces. The lipid bilayer serves as the primary medium for mechanosensing, responding to external mechanical influences through changes in shape, such as stretching, thinning, and alterations in curvature.^17^ In mammalian cells, which do not possess rigid cell walls, the cortical cytoskeleton helps preserve excess membrane area by forming structures like microvilli and membrane ruffles, thus actively managing local membrane tension and precisely regulating the cell’s sensitivity to mechanical stimuli.^17^

Within this framework, mechanosensitive ion channels, which act as molecular devices transforming membrane stress into ion movement, have emerged as a key area of study in mechanotransduction. In 2010, Coste and colleagues discovered Piezo family proteins via functional assays, confirming their role as the molecular components of mammalian MACs.^11^

### Introduction to the piezo family

The term Piezo originates from the Greek term “piezi,” which translates to “pressure.”^11^ In humans, the Piezo ion channels are produced by two separate genes, PIEZO1 and PIEZO2, found on chromosomes 16 and 18, respectively. The Piezo1 protein consists of 2,521 amino acids, while the Piezo2 protein is made up of 2,752 amino acids, both being large multi-transmembrane proteins with an approximate molecular weight of 300 kDa.^18^^,^^19^

The Piezo1 channel’s fundamental architecture consists of several repeating domains and is generally categorized into three regions: the N-terminal, the membrane domain, and the C terminal.^20^ Research utilizing cryo-electron microscopy (cryo-EM) has shown that the Piezo1 complex measures around 200 Å in diameter, featuring a central pore module along with three “blade” structures that extend outward, resembling a three-bladed propeller.^12^^,^^21^ In a study by Zhao et al., the creation of Piezo1-ASIC1 chimeric channels confirmed that the C-terminal region functions as a distinct pore module. Additionally, it was found that the extensive transmembrane domain at the N-terminal (residues 1–2,190) acts as a separate mechanotransduction module.^22^ This module includes elongated “blade” structures that can independently sense mechanical forces and facilitate the opening of the pore, thereby illustrating the molecular mechanism of Piezo1, where the “blade” serves as the mechanosensor and the “pore” functions as the effector.

**Figure 1:**
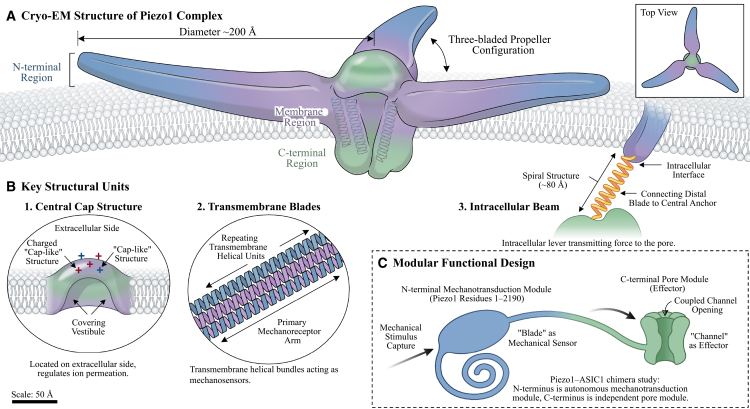
Structural architecture and mechanogating mechanism of the Piezo1 channel (A) Cryo-EM structure of the Piezo1 complex. This top view representation of the homotrimeric Piezo1 channel illustrates the characteristic three-bladed propeller configuration, with a diameter of approximately 200 Å. The structure reveals the central cap region, transmembrane domains, and the overall molecular architecture, all resolved through cryo-electron microscopy. (B) Key structural units of the Piezo1 channel. This schematic illustration highlights three major structural components: (1) the central cap structure located on the extracellular side, which covers the charged vestibule interface and regulates ion permeation; (2) the transmembrane blades, composed of repeating helical units (∼80 Å), that function as primary mechanosensors; and (3) the intracellular beam, which serves as a lever to transmit mechanical force to the pore region. (C) Modular functional design of Piezo1. This diagram depicts the functional domain organization based on Piezo1-ASIC1 chimera studies. The N-terminal mechanotransduction module (Piezo1 residues 1–2,190) operates as an autonomous mechanoreceptor that captures mechanical stimuli, while the C-terminal pore module functions as the effector domain responsible for channel opening. The “blade” structures act as mechanical sensors, and the intracellular arm connects the distal blade to the central anchor, transmitting conformational changes to gate the channel. Scale bars: 50 Å.

Channel inactivation is governed by a hydrophobic gate within the inner helix, formed by two conserved residues (L2475/V2476). Mutation to hydrophilic residues delays inactivation, confirming that hydrophobicity, rather than pore occlusion, is the primary determinant of inactivation kinetics.^23^ In light of these findings, Zheng et al. introduced a kinetic model that incorporates a hydrophobic barrier: during the inactivation process, the inner helix (IH) of Piezo1 may experience twisting and contraction. This change in conformation results in the simultaneous rotation of L2475 and V2476 toward the pore’s interior, effectively blocking ion flow by creating a high-energy hydrophobic barrier (or “vapor lock”) without fully occluding the pore (with a diameter of less than 6 Å), thus facilitating channel closure (Figure 1).^23^

The assembly configuration known as “blade-beam-pore” aligns with a “lever-like mechanism,” where membrane tension facilitates the expansion or rotation of the blade. This action enhances and transmits torque via the beam to the pore gate, ultimately leading to the opening of the cation channel. The structural and mechanistic evaluations reinforce the identification of Piezo1 as the molecular component of a mechanogated channel primarily influenced by membrane tension. In contrast, Piezo2 is predominantly found in sensory neurons and various other tissues,^24^ such as those involved in sensory perception, including dorsal root ganglia, sensory nerve endings, and Merkel cells.^25^ Given its very low expression levels and the scarcity of functional data in dermal fibroblasts, this review will concentrate on Piezo1.

### ION selectivity and Ca^2+^ permeability characteristics of Piezo1

While Piezo1 does allow some passage of Na^+^ and K^+^ ions,^26^^,^^27^ its primary function is to preferentially select for Ca^2+^, a trait that has been consistently observed in cardiomyocytes,^28^^,^^29^ vascular endothelial cells,^30^^,^^31^ and immune cells.^13^^,^^32^ This ion selectivity has been attributed, at least in part, to the channel’s distinct three-dimensional architecture, where conformational changes in the pore induced by tension are thought to interact with specific amino acid residues to dynamically modulate the affinity and permeability for Ca^2+^.^33^

## The “TRIGGER” role of calcium ions and spatiotemporal encoding

### From gating to signaling

The activation of Piezo1 channels through mechanogating represents the crucial first stage in converting physical forces into biochemical signals within cells. As a classic example of a mechanosensitive non-selective cation channel, Piezo1 reacts directly to changes in membrane tension or fluid shear stress, facilitating a swift influx of extracellular Ca^2+^. This section will explore the mechanisms by which Ca^2+^ signals are encoded in both time and space, examining how Piezo1 transforms prolonged mechanical tension into intracellular calcium transients characterized by distinct “fingerprints”, thus laying the groundwork for the precise initiation of downstream signaling pathways.

### Temporal dynamic encoding of Ca^2+^ signals

Importantly, the Ca^2+^ signaling mediated by Piezo1 is not simply a constant increase in intracellular calcium levels; it displays complex temporal dynamics. For instance, Ranade et al. found that in HEK293T cells and mouse embryonic vascular endothelial cells, laminar shear stress can directly stimulate Piezo1, leading to quickly adapting inward currents. This means the channel swiftly inactivates after it opens, resulting in brief electrical signals instead of prolonged depolarization.^34^ In a similar vein, Pathak et al. showed that in human neural stem cells, Piezo1 activated by traction forces induces spontaneous Ca^2+^ transients that occur on a timescale of seconds. These transients rely on external calcium sources and can be reversibly eliminated by EGTA (ethylene glycol-bis[β-aminoethyl ether]-N,N,N′,N′-tetra-acetic acid).^8^ These oscillations in Ca^2+^, characterized by specific frequencies and waveforms, are thought to represent a “frequency encoding” system, allowing cells to differentiate between various types and intensities of mechanical stimuli.

Recent mechanistic investigations have shown that the transient pattern of intracellular calcium levels is intricately influenced by the ongoing competition between the influx of Ca^2+^ through Piezo1 and the efflux facilitated by the plasma membrane calcium pump (PMCA).^35^ In this state of “influx-efflux” balance, effective signal peaks can only arise when the intensity of mechanical stimulation is adequate to temporarily surpass the efflux rate with the influx rate. Consequently, this unique interplay of frequency and amplitude has been proposed to act as a sophisticated “molecular code” for the potentially targeted activation of specific downstream effectors (like the CaMKK/Akt pathway), which could contribute to the specificity of mechanotransduction signals.

### Spatial compartmentalization and local microdomain regulation of Ca^2+^ signals

In addition to changes over time, the distribution of Ca^2+^ signals mediated by Piezo1 shows notable spatial variability. Research by Li et al. on human umbilical vein endothelial cells (HUVECs) demonstrated that shear stress prompts the movement and concentration of Piezo1 at the leading apical lamellipodia, which is crucial for the polarization and directional alignment of endothelial cells.^36^ Experiments that inhibited Piezo1 expression using targeted siRNA or blocked its channel function with GsMTx4 revealed a significant reduction in shear force-triggered Ca^2+^ influx, highlighting Piezo1’s essential role as a “molecular gate” in this calcium signaling process.^36^ This spatial redistribution indicates that Ca^2+^ influx is not evenly spread across the cell but is restricted to particular subcellular areas, creating localized Ca^2+^ microdomains.

It is important to highlight that the findings related to the spatial distribution of Piezo1 are mainly derived from studies on endothelial cell models. There is currently no comprehensive experimental evidence to determine if fibroblasts display comparable patterns of Piezo1 localization or if this localization influences their reaction to matrix tension. Since endothelial cells mainly react to fluid shear stress, whereas fibroblasts are more responsive to matrix tension and stiffness, the distinct mechanical environments of these two cell types could result in varying spatial behaviors of Piezo1.

### Piezo1 as a “PRECISION spatiotemporal SIGNAL ENCODER”

To conclude, current evidence suggests that Piezo1 may function as an active participant in sensing mechanical forces, potentially acting as a “precision spatiotemporal signal encoder.” It appears to modulate gating kinetics, adjust frequency, and is strategically localized within cells to transform prolonged mechanical stress into intracellular Ca^2+^ transient signals that possess distinct spatiotemporal properties. The interplay of temporal encoding (through frequency and amplitude variations) and spatial encoding (via subcellular positioning) of these calcium fluctuations lays the groundwork for the accurate activation of downstream signaling pathways, preventing non-specific signal interference and ensuring a precise conversion of mechanical stimuli into cellular fate determinations. Nonetheless, while Ca^2+^ serves as a secondary messenger, it does not directly trigger the PI3K/Akt pathway; this signal must be “interpreted” and “enhanced” by intermediary molecules like calmodulin (CaM) and calcium/CaM-dependent protein kinases (CaMKs).^37^^,^^38^^,^^39^^,^^40^ In the following sections, we will provide a detailed discussion on how these molecular intermediaries link Piezo1-induced Ca^2+^ transients to the activation of the PI3K/Akt signaling pathway.

## From ion flux to kinase cascade—construction of molecular bridges

### Overview of the PI3K/AKT signal transduction pathway

Phosphoinositide 3-kinases (PI3Ks) are categorized into three main classes: I, II, and III, with class I further split into subclasses IA and IB. Class IA PI3Ks consist of a catalytic component (either p110α, p110β, or p110δ) paired with a regulatory component, which may include p85α and its variants (p55α, p50α), p85β, or p55γ.^41^ In contrast, class IB PI3Ks are also heterodimers, featuring p110γ as the catalytic unit alongside regulatory units p101 or p84.^41^ This discussion centers on class I PI3K, whose regulatory subunit is equipped with SH2 and SH3 domains that selectively bind to targets containing phosphotyrosine. The functionality of this class is mainly influenced by receptor tyrosine kinases (RTKs) and G protein-coupled receptors (GPCRs).^42^

In response to signals from upstream, PI3K is brought to the plasma membrane where it facilitates the conversion of PIP_2_ into PIP_3_ through phosphorylation. This newly formed PIP_3_ selectively attracts PDK1 and Akt to the membrane via their PH domains. Following this, Akt undergoes complete activation through dual phosphorylation at the Thr308 site by PDK1 and at the Ser473 site by mTORC2, which triggers subsequent signaling pathways.^43^

p110α and p110β are the major expressed class IA isoforms, whereas p110δ and p110γ are mainly restricted to hematopoietic lineages.^43^^,^^44^ Conte et al. demonstrated that selective inhibition of the p110α isoform, but not p110δ, blocks TGF-β-induced myofibroblast differentiation of lung fibroblasts.^45^ Similarly, we propose that p110α may be the main catalytic subunit mediating Piezo1-Ca^2+^ driven PI3K activation in skin fibroblasts.

### Molecular mechanisms of Ca^2+^-PI3K/Akt coupling

Understanding how significant local Ca^2+^ influx interacts with the PI3K/Akt signaling pathway is crucial and warrants further investigation, as it underpins all related concerns. While the exact molecular processes through which Piezo1-mediated Ca^2+^ influx triggers PI3K/Akt activation are still under study, current findings indicate that the coupling between Ca^2+^ and the PI3K/Akt pathway could occur via several concurrent or sequential routes.

Before proceeding to detailed mechanistic discussion, we provide a concise evidence roadmap (Table 1) to orient the reader. Each candidate molecular bridge is assessed along three dimensions: (i) biochemical mechanism—whether the core enzymatic or binding reaction has been demonstrated in any cell system; (ii) dermal fibroblast evidence—whether the pathway has been specifically validated in skin fibroblasts under mechanical stimulation; and (iii) wound-healing functional relevance—whether perturbation of the pathway has been shown to affect fibroblast behaviors (proliferation, migration, differentiation, or matrix synthesis) during wound repair. As summarized below, only the integrin-FAK bridge currently satisfies all three criteria in dermal fibroblasts. The remaining bridges carry strong biochemical rationale but require cell-type-specific experimental confirmation, a gap we highlight throughout the following subsections and revisit in the comprehensive validation matrix (Table 2).

**Table 1:** Evidence roadmap for the four candidate molecular bridges linking Piezo1-Ca^2+^ TO PI3K/Akt in dermal fibroblasts

Molecular bridge	Biochemical mechanism	Evidence in dermal fibroblasts	Wound-healing functional relevance	Confidence level	Key gaps
① CaM/CaMK → PI3K/Akt (Section 3.3)	**Established.** pY99-CaM-p85 binding demonstrated biochemically (Zhang et al. 2017); CaMKK→Akt confirmed in Non fibroblast system systems (Tokumitsu 2022; Zheng 2022).	**Not validated.** Upstream kinase for CaM(Y99) identified only in KRAS-mutant cancer cells; no fibroblast-specific data under mechanical stimulation.	**Indirect.** Piezo1→Ca^2+^→Akt link shown (Huang 2025; Zhan 2024), but CaM/CaMK as the intermediary has not been isolated in fibroblast wound models.	◐ Plausible but unverified	Identify the kinase responsible for CaM (Y99) phosphorylation in mechanically stimulated fibroblasts; co-IP of pY99-CaM with p85 under stretch.
② Integrin-FAK → PI3K/Akt (Section 3.4)	**Established.** FAK(Y397) → p85 recruitment→PI3K activation is a canonical signaling pathway (Xia 2004).	**Directly supported.** Piezo1 localizes to focal adhesions in HFF (Yao 2022); Piezo1-dependent Ca^2+^ enhances FAK(Y397) phosphorylation (Chen 2023); FAK→PI3K validated in dermal fibroblasts (Li 2016).	**Directly supported.** PI3K inhibition (LY294002) blocks fibroblast migration and wound contraction *in vivo* (Li 2016); Piezo1 knockdown reduces Akt phosphorylation in keloid fibroblasts (Zhang 2024).	● Best-validated pathway	Complete chain not yet demonstrated as a continuous, real-time sequence in a single fibroblast experiment.
③ Calpain ⊣/→ PI3K/Akt (Section 3.5)	**Established in other contexts.** Moderate calpain sustains Akt via PP2A degradation (Ho et al. 2012); excessive calpain cleaves PI3K (Beltran et al. 2011).	**Not tested.** No study has examined calpain activation downstream of Piezo1 in dermal fibroblasts.	**Not tested.** Biphasic Ca^2+^-calpain model is extrapolated from mammary carcinoma and auditory system models.	○ Speculative	Measure calpain activity and PP2A/PI3K cleavage products in fibroblasts under graded Piezo1 stimulation (e.g., dose-response to Yoda1).
④ Panx1-ATP-P2Y2 → PI3K/Akt (Section 3.6)	**Established in RBC/endothelial cells.** Piezo1→PANX1→ATP release (Cinar 2015); P2Y2→FAK/Akt via RGD-integrin route (Sathanoori 2017).	**Not demonstrated.** PANX1 is expressed in fibroblasts and P2Y2 expression confirmed (Solini 2003), but Piezo1→PANX1→ATP release has not been shown in dermal fibroblasts.	**Indirect.** P2Y2 antagonism inhibits keratinocyte wound closure (McEwan 2021); P2Y2 knockout impairs fibroblast function in skeletal muscle (Chen 2021); not tested in dermal wound context.	◐ Plausible but unverified	Quantitative ATP release measurement from dermal fibroblasts following Yoda1 ± PANX1 inhibition; P2Y2 antagonist (AR-C118925XX) effect on Piezo1-induced Akt phosphorylation.

**Note:** confidence grading: ● = directly supported by experimental evidence in dermal fibroblasts; ◐ = biochemically plausible with supporting evidence from other cell types, but unverified in dermal fibroblasts; ○ = speculative, based on mechanistic analogy with no fibroblast-specific data.

“Key Gaps” column highlights the minimum experiments required for elevation to the next confidence level. For detailed per-step validation across multiple cell types, see Table 2.

Abbreviations: FB, fibroblast; HFF, human foreskin fibroblast; RBC, red blood cell; co-IP, co-immunoprecipitation; HTS, hypertrophic scar; MEF, mouse embryonic fibroblast.

**Table 2:** Validation status of Piezo1-Ca^2+^-PI3K/Akt signaling mechanisms across cell types

Signaling mechanism/pathway	Dermal FB	Lung FB	Cardiac FB	Vascular EC	Key references
**I. Piezo1 basic mechanisms**					

1.1 Piezo1 channel expression	★★★	★★★	★★☆	★★★	Coste 2010; Yao 2022
1.2 Piezo1-Ca^2+^ influx	★★★	★★☆	★★☆	★★★	Pathak 2014; Li 2014
1.3 Piezo1 focal adhesion localization	★★★	–	–	–	Yao 2022
1.4 CADM1 prolongs Piezo1 opening	★★☆	–	–	–	Koster 2024

**II. Ca**^**2+**^**-PI3K/Akt coupling mechanisms**					

2.1 CaM-pY99-PI3K pathway	★☆☆	★☆☆	★☆☆	★☆☆	Zhang 2017
2.2 CaMKK-Akt direct activation	★☆☆	★☆☆	★☆☆	★☆☆	Anderson 1998
2.3 CaMKII-PI3K/Akt	★☆☆	–	★★☆	★★★	Tokumitsu 2022;Zheng 2022
2.4 Integrin-FAK-PI3K axis	★★★	★★☆	★★☆	★★☆	Chen 2023; Yao 2022
2.5 Calpain regulation	★☆☆	★☆☆	★☆☆	★☆☆	Ho 2012
2.6 ATP-P2Y2 paracrine pathway	★☆☆	–	★★☆	★★★	Cinar 2015; McEwan 2021

**III. Fibroblast functional regulation**					

3.1 Piezo1-Akt-proliferation	★★★	–	–	★★★	Zhang 2024; Xu 2022
3.2 Piezo1-Akt-GSK3β-CyclinD1	★☆☆	–	–	–	Xu 2022
3.3 Piezo1-FAK-migration	★★★	–	–	★★★	Chen 2023; He 2021; Li 2016
3.4 Piezo1 spatial localization-migration	★★☆	–	–	★★★	Li 2014; Yang 2022
3.5 TGF-β/Smad-myofibroblast differentiation	★★☆	★★★	★★☆	–	Xu 2025; Conte 2011
3.6 PI3K/Akt-Smad3 synergy	★★★	★★★	–	–	Li 2016; Luo 2017
3.7 mTOR-collagen synthesis	★★★	–	–	–	Byun 2024
3.8 Metabolic reprogramming (glycolysis)	★★★	–	–	–	Xue 2025
3.9 Arginine-proline metabolic axis	★★★	–	–	–	He 2023

**IV. Pathology-related mechanisms**					

4.1 Keloid-Piezo1 overexpression	★★★	–	–	–	Zhang 2024
4.2 Stiffness-Piezo1 positive feedback	★☆☆	★★☆	–	–	Li 2022
4.3 Macrophage-fibroblast mechanical contact	★★★	★★★	–	–	Ezzo 2024

Note: ★★★ = direct validation (clear experimental evidence directly supporting this mechanism in this cell type); ★★☆ = indirect validation (related experimental evidence but not directly targeting this mechanism); ★☆☆ = inference (reasonable inference based on mechanistic similarity from other cell types); — = no evidence, may be the focus of future research FB = fibroblast; EC = endothelial cell.

### CAM/CAMKK-mediated direct activation

#### CAM-PY99 pathway

CaM serves as the main mediator and signal transducer for calcium ions in eukaryotic cells.^46^^,^^47^ When calcium ions (Ca^2+^) attach to CaM, it triggers a significant phosphorylation modification at the Tyr99 position, resulting in the formation of high-affinity pY99-CaM. This phosphorylated form of CaM has been proposed to mimic the activation mechanism of RTKs, potentially allowing it to bind to the SH2 domain of the class I PI3Kα regulatory subunit, p85. It has been suggested that this interaction may lead to allosteric changes that could relieve the autoinhibition of the p85 subunit on the p110 catalytic subunit, thereby contributing to disinhibition. As a consequence, the catalytic activity of p110 is activated, facilitating the production of phosphatidylinositol-3,4,5-trisphosphate (PIP_3_), which subsequently recruits and activates the downstream effector molecule Akt through phosphorylation.^48^^,^^49^

Research has demonstrated a strong interconnection between Piezo1 and the PI3K/Akt signaling pathway. For instance, in models induced by IL-1β, Piezo1 has been shown to function as an upstream regulator of the PI3K/Akt/mTOR signaling pathway, playing a crucial role in its regulation.^50^ While the effects of Piezo1 vary across different tissues and cell types, its role as an “upstream initiator” of the PI3K/Akt pathway is largely consistent. Recent investigations in actively dividing cell models have shown that pharmacological stimulation of Piezo1, such as with Yoda1, can trigger notable intracellular Ca^2+^ spikes and significantly enhance the phosphorylation of Akt and its downstream target mTOR through calcium-dependent pathways.^51^ This evidence raises the possibility that the Piezo1-Ca^2+^-Akt axis could represent a candidate mechanotransduction mechanism, although direct confirmation in dermal fibroblasts is still required.

In addition, Huang and colleagues demonstrated that utilizing nanotopographic structures to mimic physical microenvironments can markedly boost the activity of Piezo1 channels and induce increases in intracellular calcium levels. A pivotal finding of this research was the application of the Piezo-specific inhibitor GsMTx4, which allowed the researchers to directly observe notable decreases in the levels of downstream p-PI3K and *p*-Akt, thereby confirming the molecular regulatory pathway of “Piezo-Ca^2+^-PI3K/Akt.”^52^

A key gap in this pathway involves the identity of the upstream tyrosine kinase responsible for CaM Tyr99 phosphorylation in the context of mechanotransduction. Current biochemical evidence mainly comes from cancer cell models, indicating that Src family kinases are the main mediators of CaM Y99 phosphorylation.^53^ Zhang et al. demonstrated that phosphorylation of this residue can bind with high affinity to the p85-SH2 domain, but the kinase-substrate relationship was established in KRAS-mutant cancer cells.^48^ Therefore, we point out (i) the specific kinase responsible for CaM(Y99) phosphorylation has been identified mainly in KRAS-mutant cancer cells, but remains undefined in mechanically stimulated fibroblasts; and (ii) whether this phosphorylation event requires concurrent RTK activity has not been tested; therefore, we classify the CaM-pY99-PI3K bridge in the context of dermal fibroblasts as mechanistically plausible but experimentally unverified (Table 2). Critical validation experiments would include (i) immunoprecipitation of pY99-CaM with p85 in fibroblasts subjected to mechanical stretch; and (ii) real-time monitoring of CaM phosphorylation levels using FRET-based biosensors in fibroblasts plated on substrates of varying stiffness.

#### CAMKK-Akt direct pathway

CaMK is a serine/threonine kinase that becomes activated upon its interaction with CaM, a key player in calcium signal transduction.^54^^,^^55^ When extracellular signals trigger an increase in intracellular calcium ion (Ca^2+^) levels, CaM binds to these ions, leading to structural changes that affect the regulatory region of CaMKs. This allosteric modulation shifts the CaMK catalytic domain from an autoinhibited form to an active configuration.^56^ Subsequently, it associates with and activates CaMKKβ/2, a variant of CaMK kinase (CaMKK),^57^^,^^58^ which in turn directly stimulates Akt.^59^ This indicates that the activation of the PI3K/Akt signaling pathway may occur independently of PI3K, allowing for direct activation of Akt and influencing downstream targets.

#### CAMKII-PI3K/Akt

The activation of Piezo1 leads to a swift increase in the concentration of intracellular Ca^2+^. This calcium acts as a secondary messenger, attracting CaM and triggering the activation of CaMKII, which sets off a series of downstream kinase reactions.^37^^,^^39^^,^^40^ Previous research, along with recent observations in vascular endothelial cell models, indicates that the phosphorylation of PI3K can occur through the autophosphorylation of CaMKII.^59^^,^^60^ This raises the possibility that the influx of Ca^2+^ induced by mechanical forces may serve as a signaling molecule, potentially facilitating the recruitment of CaM and the binding of CaMKII, which has been proposed to phosphorylate PI3K under certain conditions. This process leads to the phosphorylation of Akt and the subsequent activation of mTOR, ultimately promoting protein synthesis and the maintenance of organelle homeostasis.

The results provide compelling evidence for the idea that analogous processes could be present in dermal fibroblasts. Specifically, mechanical stress, facilitated by Piezo1-induced calcium entry, serves as a crucial secondary messenger that directly stimulates the activation of the PI3K/Akt pathway responsible for cell survival and growth. However, we prefer to classify the discussion of the above pathways in skin fibroblasts as inferential evidence (Table 2), because there is no clear experiment and research to prove that these pathways are found and verified in fibroblasts.

## Piezo1 cooperates with integrins—PI3K/Akt

### Integrin-focal adhesion: Molecular platform for mechanosensing

Cells detect and transform mechanical stimuli through two primary types of mechanosensors: stretch-activated ion channels and focal adhesion complexes.^61^^,^^62^ Focal adhesions, which are centered around integrins, bring together various scaffold proteins and signaling molecules to create platforms for mechanochemical transduction. Integrins are transmembrane receptors made up of α and β subunits; their extracellular regions interact with components of the ECM like collagen, laminin, and tenascin. Meanwhile, their intracellular regions attract scaffold proteins such as Talin and Paxillin, as well as kinases like FAK and Src, facilitating the transmission of mechanical stress into the cell. This process is crucial for regulating cell adhesion, migration, proliferation, and differentiation.^63^ Recent research has indicated a coordinated spatiotemporal relationship between local calcium signals and focal adhesions,^64^ implying that mechanosensitive ion channels might play a role in the signal transduction mediated by focal adhesions.

### Force-dependent localization of Piezo1 at focal adhesions

The conventional perspective suggests that integrins serve as the main detectors for sensing the stiffness of the ECM.^65^^,^^66^ Nevertheless, research by Yao et al. revealed that Piezo1 is located in focal adhesions and plays a role in how fibroblasts perceive ECM stiffness.^67^ Their findings indicated that as human foreskin fibroblasts (HFFs) spread, Piezo1 progressively accumulated at established focal adhesions at the tips of stress fibers within 30–60 min. Fluorescence recovery after photo bleaching studies indicated that Piezo1 had a significantly longer residence time at focal adhesions (t_½_ ≈ 120 s) compared to paxillin (t_½_ ≈ 25 s) and integrin β3 (t_½_ ≈ 90 s), implying that it forms stable associations with essential components of focal adhesions. Importantly, this research also showed that the localization of Piezo1 is dependent on the continuous mechanical tension of focal adhesion elements.^67^

Further investigations into the structure and function demonstrated that the linker domain of Piezo1, spanning amino acids 1,418 to 1,656, plays a crucial role in facilitating interactions with focal adhesions.^67^ This suggests that Piezo1 serves not only as a standalone mechanosensitive channel but also as an integral part of the focal adhesion complex.

### The Piezo1-Ca^2+^-FAK-PI3K/Akt signaling axis

Research has indicated that integrin signaling commonly triggers the activation of the PI3K/Akt pathway.^68^^,^^69^^,^^70^ Investigations into Piezo1 have demonstrated its localization to focal adhesion sites during the process of cell spreading, suggesting a potential physical link to PI3K/Akt signaling.^71^ A study by Chen et al. highlighted the synergistic function of Piezo1 in this mechanism: integrins serve as physical anchors that detect matrix stiffness and facilitate the development of cytoskeletal tension, while Piezo1 functions as a secondary sensor for this tension. Notably, the findings indicated that the influx of Ca^2+^ through Piezo1 may significantly enhance the phosphorylation levels of FAK (Y397) in the cell types studied.^72^

Existing research has suggested that FAK may activate the PI3K/Akt signaling pathway,^73^ which raises the hypothesis of a potential signaling axis involving Piezo1, Ca^2+^, FAK, and PI3K/Akt that warrants direct validation in dermal fibroblasts. To address the issues in this pathway, we propose a model based on currently available evidence and annotate the validation status of each step in fibroblasts.

1. Mechanical load (matrix stiffness or tension) increases membrane tension → Piezo1 channel opens → Ca^2+^ influx. Local Ca^2+^ elevation enhances FAK autophosphorylation. Partially validated Chen et al. demonstrated that Piezo1-dependent Ca^2+^ influx enhances FAK phosphorylation in fibroblasts,^72^ but the specific biochemical mechanism between Ca^2+^ and FAK remains unclear.
2. Phosphorylated FAK (Y397) recruits the p85 regulatory subunit of PI3K via SH2 domain interaction at the plasma membrane.^74^ The integrin-FAK → PI3K connection has been validated in fibroblasts; the Piezo1 → FAK → PI3K as a continuous sequence has not been confirmed.
3. PIP_3_ binds to PDK1 and Akt → Akt phosphorylation.^75^ Phosphorylation of Akt downstream of Piezo1 is verified in fibroblasts.

It should be noted that this temporal model is reconstructed from separate studies and has not been verified as a continuous, real-time sequence within a single fibroblast experiment.

### CADM1 prolongs Piezo1 activation: A key mechanism for maintaining calcium signals

In their 2024 study, Koster and colleagues utilized proximity labeling methods to chart the surface interactome of Piezo1, revealing that the cell adhesion molecule CADM1 plays a crucial role in regulating Piezo1. The interaction occurs via CADM1’s transmembrane domain, which notably prolongs the open state of Piezo1 and delays its inactivation.^76^ This discovery offers insights into the potential mechanisms by which Piezo1 sustains prolonged Ca^2+^ signaling, thereby activating the FAK and PI3K/Akt pathways.

Drawing from current research, a putative positive feedback mechanism can be tentatively outlined: in rigid matrices, the contractile force driven by myosin II may be amplified, which raises membrane tension via the PI3K/PIP_3_ signaling pathway, potentially leading to the activation of Piezo1 channels, although this model requires experimental validation as a continuous sequence. The opening of Piezo1 facilitates the influx of Ca^2+^, which is sustained over longer durations under the influence of CADM1, subsequently triggering the FAK/RhoA/ROCK signaling cascade, which further boosts the contractile force of myosin II. This process may clarify how fibroblasts can sustain an activated state on rigid matrices and differentiate into myofibroblasts.^67^^,^^76^

Based on the evidence presented, we suggest the following hypothesis: mechanical signals attract Piezo1 to focal adhesions, where it works alongside integrins to detect the stiffness of the ECM. This interaction leads to a Ca^2+^ influx mediated by Piezo1, which activates the PI3K/Akt pathway via FAK. Additionally, CADM1 plays a role in prolonging the open state of Piezo1, ensuring the continuity of the Ca^2+^ signal. This signaling pathway, termed “integrin-Piezo1-Ca^2+^-FAK-PI3K/Akt,” may represent a fundamental component of mechanotransduction in dermal fibroblasts. This represents the best-validated pathway in dermal fibroblasts (Table 2), However, the complete signaling chain has not yet been demonstrated as a continuous, temporally resolved sequence within a single fibroblast experiment. So, we present this as a testable dependency: if CADM1 expression is low or absent in dermal fibroblasts, the sustained Ca^2+^ input required for the integrin-FAK-PI3K/Akt feedback loop would need alternative mechanisms.

Nonetheless, this theory encounters several significant challenges that need to be addressed: (1) the arrangement of Piezo1 and integrins at focal adhesions on a nanoscale level and the alterations in their dynamics; (2) the precise manner in which Ca^2+^ signals modulate the activation of FAK and PI3K/Akt in both temporal and spatial contexts; (3) the molecular pathways through which CADM1 influences the inactivation of Piezo1 and its preservation across various cell types; and (4) the need for functional confirmation of this signaling pathway in both normal and disease states, including skin wound healing and fibrosis. Tackling these challenges will enhance our understanding of the molecular processes involved in mechanosignal transduction and may reveal new therapeutic targets for conditions like fibrosis.

## Regulatory mechanism: The dual role of calpain

Calpains are a type of cysteine protease that relies on Ca^2+^^77^^,^^78^ and exhibit context-sensitive functions in the regulation of the PI3K/Akt signaling pathway. In normal physiological states, a balanced level of calpain activity has been reported to sustain the phosphorylation of Akt, at least in part through promoting the breakdown of PP2A (protein phosphatase 2A).^79^ Conversely, during conditions of stress or excessive calcium, overactive calpains can directly target and cleave the subunits of PI3K, resulting in the deactivation of the signaling pathway,^80^^,^^81^but this has not been directly demonstrated in dermal fibroblasts.

The dual function implies that the strength of Ca^2+^ signals mediated by Piezo1 could influence how calpains affect the PI3K/Akt pathway—intermediate Ca^2+^ fluctuations might promote pathway activation, whereas prolonged calcium excess could initiate negative feedback. The role of calpain in modulating Piezo1-PI3K/Akt signaling is classified as inference-level (Table 2). No study has directly examined calpain activation downstream of Piezo1 in dermal fibroblasts, nor has the biphasic regulation model been validated in this cell type. This hypothesis is extrapolated from studies in mammary carcinoma^79^ and auditory systems,^81^ and should be considered speculative pending direct experimental confirmation.

## Paracrine mechanism: The ATP-P2Y2 pathway

### Molecular basis of Piezo1-mediated ATP release

The release of ATP following the activation of Piezo1 does not happen directly through the ion channel but relies on subsequent Ca^2+^ effectors. Research indicates that the Ca^2+^ influx initiated by Piezo1 can stimulate pannexin-1 (PANX1) channels, which are large-pore, non-selective channels gated by Ca^2+^ that allow ATP and other small molecules (less than 1 kDa) to exit the cytoplasm into the extracellular environment. Cinar et al. were the first to show in human red blood cells that Piezo1 acts as the primary receptor for ATP release induced by mechanical forces, with PANX1 serving as the principal channel for ATP outflow; inhibiting either Piezo1 or PANX1 pharmacologically led to a reduction of about 50% in ATP release triggered by shear forces.^35^

It is important to note that Piezo1 and PANX1 might create functional or even physical complexes. Research by Desplat et al. demonstrated through co-immunoprecipitation and pull-down assays in mouse cholangiocytes that there are direct interactions between Piezo1 and PANX1, which co-localize at the cell membrane.^82^ The presence of these “mechanoreceptor-ATP release channel” complexes offers a solid molecular foundation for Piezo1-driven paracrine signaling. Comparable pathways involving Piezo1, PANX1, and ATP release have also been identified in endothelial cells,^83^ which are sensitive to mechanical stimuli, indicating that this mechanism of coupling mechanical force to ATP release is likely conserved. Nonetheless, there are some limitations: the understanding of the Piezo1-PANX1-ATP release mechanism is mainly derived from studies on red blood cells, endothelial cells, and cholangiocytes. There is currently insufficient research directly examining Piezo1-mediated ATP release in dermal fibroblasts. However, since PANX1 is broadly expressed in fibroblasts and is involved in various ATP-dependent cellular processes, it is plausible to hypothesize that this mechanism may also be present in fibroblasts.

### Multiple connections between P2Y2 receptors and PI3K/Akt

ATP released outside the cell transforms mechanical stimuli into intracellular biochemical pathways through the activation of P2Y2 receptors, which are coupled with Gq/11 G proteins.^84^ This indicates several links between P2Y2 and the PI3K/Akt signaling pathway.

1 Traditional Gq signaling route: stimulation of P2Y2 → Gq → PLCβ → production of IP_3_ → release of Ca^2+^ from the endoplasmic reticulum → activation of PI3K/Akt.^83^
2 The P2Y2-integrin-FAK pathway: among the limited GPCRs, P2Y2 receptors possess an RGD (Arg-Gly-Asp) domain that binds integrins.^85^ Research by Erb et al. demonstrated that the RGD motif located in the first extracellular loop of P2Y2 can engage directly with αVβ3/β5 integrins; altering RGD to RGE resulted in a roughly 1,000-fold increase in the concentration of agonist needed to stimulate FAK and ERK activation.^85^ Additionally, Sathanoori et al. validated that in HUVECs, the phosphorylation of FAK(Y397), Akt(S473), and eNOS(S1177) induced by shear stress is reliant on the RGD region of P2Y2.^86^

This discovery carries significant consequences: it suggests that P2Y2 not only stimulates PI3K/Akt via the traditional GPCR-Gq-Ca^2+^ route but also collaborates with pathways that sense mechanical forces directly, utilizing the RGD-integrin-FAK framework. Essentially, ATP released by Piezo1 may “merge” through P2Y2 into the Integrin-FAK-PI3K pathway, which has been confirmed in fibroblasts, thereby offering mechanisms for both chemical amplification and spatial distribution of mechanical force signals.

### Evidence for purinergic signaling in cutaneous wound healing

In the process of skin wound healing, ATP functions as a “damage-associated molecular pattern” that is swiftly released from injured cells after trauma, with local levels possibly increasing from around 10 nM in healthy tissue to micromolar concentrations. This ATP outside the cell is crucial for the repair of wounds as it stimulates P2Y receptors.^87^

Keratinocytes: in a study by McEwan et al. utilizing a HaCaT cell scratch injury model, it was demonstrated that following an injury, ATP is released via PANX1 channels, which then activates P2Y2 receptors and triggers the PLC/IP_3_ signaling pathway to facilitate wound healing. The use of the P2Y2-specific antagonist AR-C118925XX notably hindered the healing process.^88^ Importantly, the findings indicated that P2Y2 primarily enhances cell migration rather than proliferation, aligning with the needs of early re-epithelialization during wound healing.

Human dermal fibroblasts are known to express various subtypes of P2 receptors, such as P2X3, P2X4, P2X7, and P2Y1, P2Y2, P2Y4, and P2Y6.^89^ Research by Chen et al. demonstrated that the absence of P2Y2 in skeletal muscle fibroblasts led to a notable decrease in the phosphorylation of Akt, ERK, and PKC, as well as a reduction in fibroblast proliferation, migration, and their transformation into myofibroblasts, which was indicated by lower levels of α-SMA, collagen I, and CTGF.^90^ Lu et al. also found that in cardiac fibroblasts, ATP enhances collagen production by around 60% via the P2Y2-ERK signaling pathway.^91^

Models of skin fibrosis: research by Perera et al. indicated that in studies of systemic sclerosis, the release of ATP from skin fibroblasts was elevated in hypoxic environments. Additionally, extracellular ATP notably boosted the production of IL-6 and collagen I via the P2Y2-p38 signaling pathway. The P2Y2 antagonist AR-C118925XX showed considerable anti-fibrotic properties in a mouse model of skin fibrosis induced by bleomycin.^92^

To conclude, the paracrine role of ATP-P2Y2 may act as a “chemical relay hub” for the Piezo1-PI3K/Akt signaling pathway: activation of Piezo1 leads to Ca^2+^ influx, which triggers PANX1 channels to open, resulting in ATP release that activates P2Y2. This activation can initiate two pathways: (1) Gq → Ca^2+^ → PI3K/Akt and (2) RGD-integrin-FAK → PI3K/Akt. Such a design, integrating multiple pathways, enhances the stability of signals and allows for broader spatial influence within the system. Even if the activation of Piezo1 in a single cell is brief and localized, the ATP released can influence adjacent cells through paracrine signaling, leading to synchronized responses at the tissue level.

The Piezo1-PANX1-ATP-P2Y2 mechanism is classified as inference-level (Table 2) for dermal fibroblasts. While PANX1 is broadly expressed in fibroblasts and P2Y2 receptor expression has been confirmed in human dermal fibroblasts,^84^ no study has directly demonstrated that Piezo1 activation triggers PANX1-dependent ATP release in this cell type. Key experiments needed in future research include (i) quantitative ATP release measurement from dermal fibroblasts following Yoda1, with/without PANX1 inhibition and (ii) assessment of whether P2Y2 antagonism (AR-C118925XX) attenuates Piezo1-induced Akt phosphorylation in fibroblasts.

## Summary of the four mechanisms mentioned

**Figure 2:**
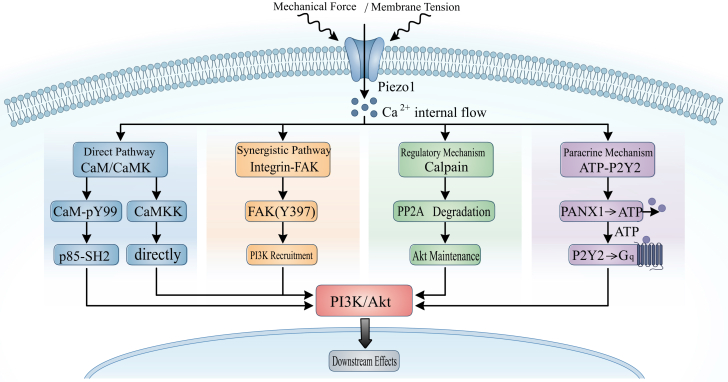
Molecular bridges coupling Piezo1-mediated Ca^2+^ influx to PI3K/Akt activation This schematic illustration delineates four distinct molecular pathways that transduce Piezo1-mediated Ca^2+^ signals to the PI3K/Akt signaling hub in fibroblasts. Direct pathway (CaM/CaMK): mechanical force induces membrane tension, which leads to the opening of the Piezo1 channel and subsequent Ca^2+^ influx. Intracellular Ca^2+^ binds to calmodulin (CaM), triggering phosphorylation at Tyr99 to produce pY99-CaM, which directly recruits PI3K by binding to the p85-SH2 domain. Alternatively, the Ca^2+^/CaM complex activates CaMKK, which can phosphorylate Akt independently of PI3K. Synergistic pathway (integrin-FAK): Piezo1 localizes to focal adhesions and collaborates with integrins to sense ECM stiffness. The Ca^2+^ influx through Piezo1 enhances phosphorylation of FAK at Y397, which subsequently recruits and activates PI3K, culminating in Akt activation. Regulatory mechanism (calpain): Ca^2+^-dependent calpain proteases exhibit a dual regulatory role in PI3K/Akt signaling. Under physiological conditions, moderate calpain activity facilitates the maintenance of Akt through the degradation of PP2A (protein phosphatase 2A). Conversely, excessive Ca^2+^ levels can activate calpain-mediated degradation of PI3K, serving as a negative feedback mechanism. Paracrine mechanism (ATP-P2Y2): activation of Piezo1 triggers the Ca^2+^-dependent opening of pannexin-1 (PANX1) channels, leading to ATP release into the extracellular space. The released ATP activates P2Y2 receptors coupled to Gq proteins, which amplifies intracellular Ca^2+^ signals and further promotes PI3K/Akt activation via both classical GPCR signaling and integrin-mediated pathways.

The four mechanisms mentioned exhibit different levels of validation in dermal fibroblasts (Figure 2). At present, the integrin-FAK pathway stands out as the sole mechanism backed by direct experimental evidence in fibroblasts. Research conducted by Yao et al.) and Chen et al. demonstrated the presence of Piezo1 at the focal adhesions of fibroblasts and its role in promoting FAK phosphorylation. As anticipated in the evidence roadmap (Table 1), the four bridges exhibit markedly different validation levels in dermal fibroblasts.

Conversely, while the mechanisms and substantial evidence for the CaM-pY99-PI3K pathway are well-established, they mainly derive from laboratory reconstitution studies and cancer cell models. Is the functioning of this pathway comparable in fibroblasts? Are fibroblast-specific regulatory elements influencing the binding of pY99-CaM to p85? These inquiries remain to be directly confirmed.

The functions of calpains and the ATP-P2Y2 signaling pathway in fibroblasts remain largely ambiguous, leading to only speculative conclusions drawn from mechanistic parallels. Upcoming studies could concentrate on: (1) real-time observation of CaM phosphorylation levels in fibroblasts; (2) measuring ATP release quantitatively after Piezo1 activation; and (3) investigating the impact of specific pathway inhibitors on the proliferation and migration of fibroblasts.

By synthesizing the aforementioned analyses, we propose that Ca^2+^ signals mediated by Piezo1 may converge at the PI3K/Akt signaling node via several parallel routes, although the degree to which each route contributes in dermal fibroblasts varies considerably in terms of direct experimental support (see Table 2 for systematic evidence grading),and, in order to provide a clear level of evidence, we summarize the verification status of each molecular bridge from three dimensions (Table 2): mechanism verification (whether biochemical reactions have been proved?), cell type specificity (has it been proved in skin fibroblasts?), and functional relevance (whether the disturbance has been proved to affect the wound healing behavior of fibroblasts?). Only the integrin-FAK pathway can be basically satisfied in all three dimensions. The CaM/CaMK pathway has strong mechanism evidence, but there are deficiencies in fibroblast-specific verification. Calpain and ATP-P2Y2 pathways are still mainly hypothetical in the context of fibroblasts. We emphasize that the multi-bridge model is a working framework designed to guide future experimental priorities, not a statement of established facts.

## Fine regulation of fibroblast fate by the PI3K/Akt pathway

### Cell proliferation

At the heart of tissue regeneration is the need to restore and sustain cell populations. In the process of skin wound healing, fibroblasts are required to survive amid the inflammatory environment while also starting proliferation processes to address tissue gaps. Increasing research suggests that the Piezo1-PI3K/Akt signaling pathway is crucial in regulating this mechanism.

In the realm of skin wound healing, significant direct evidence was presented by Zhang et al. Their research identified a specific subset of fibroblasts marked by CD74 that is particularly responsive to mechanical stretching within keloid tissue. The application of mechanical forces activated Piezo1 channels on the membranes of these cells, leading to an influx of calcium ions and a notable increase in Akt phosphorylation. This cascade involving Piezo1 and Akt was identified as a potential contributor to fibroblast growth in this specific keloid model. Furthermore, the study demonstrated that silencing Piezo1 with siRNA effectively inhibited both the stretch-induced Akt phosphorylation and fibroblast proliferation, thereby confirming the direct relationship of “Piezo1 → Akt → proliferation” in dermal fibroblasts.^75^

The precise molecular pathways through which the Piezo1-PI3K/Akt signaling pathway enhances cell survival in dermal fibroblasts remain underexplored. However, insights from studies in other tissues offer a valuable mechanistic context. In models focused on bone tissue maintenance, the activation of Piezo1 triggers the phosphorylation of PI3K and Akt via calcium-dependent pathways, leading to an increase in the anti-apoptotic protein Bcl-2 while decreasing pro-apoptotic proteins such as Bax and cleaved caspase-3, which significantly boosts cell survival under stress.^93^ In vascular endothelial cells, the targeted deletion of Piezo1 resulted in about a 60% decrease in the levels of p-PI3K and *p*-Akt, along with a notable drop in the proliferation marker PCNA.^94^ Given the strong conservation of the PI3K/Akt-Bcl-2 anti-apoptotic pathway across eukaryotic cells, it may be speculated that similar mechanisms could operate in dermal fibroblasts, pending direct experimental confirmation.

### Downstream molecular mechanisms

In their 2025 study, Qu and Zhang introduced the insightful “calcium signal fingerprint” theory, which explores the role of PI3K/Akt in regulating cell cycle progression and influencing cell growth. They emphasized that the manner in which calcium ions enter through Piezo1 influences the specificity of subsequent signaling pathways. Notably, a prolonged and gradual increase in calcium levels, often observed in cells experiencing continuous tension, is likely to selectively activate the PI3K/Akt/mTOR signaling pathway.^95^

Upon activation of the Akt/mTOR pathway, it functions as a key regulator of the cell cycle. Research by Xu et al. identified a comprehensive signaling pathway in immune cells sensitive to mechanical stimuli: when Piezo1 is activated by mechanical forces, there is a notable increase in Akt phosphorylation. This activated Akt then phosphorylates and inhibits GSK3β, which normally degrades cyclin D1. The inhibition of GSK3β results in the stable accumulation and nuclear translocation of cyclin D1, thus promoting cell cycle progression directly.^96^

The initial understanding of this mechanism was developed in immune cells, but subsequent findings indicate that it functions similarly in dermal fibroblasts: (1) the PI3K/Akt pathway is a well-preserved regulatory system for cell proliferation across eukaryotic organisms, confirmed in various cell types, including fibroblasts^97^; (2) direct evidence of the Piezo1-Ca^2+^-PI3K/Akt signaling pathway has been documented in fibroblasts (Table 1); and (3) the proliferation effect linked to Piezo1, as reported by Zhang et al. in keloid fibroblasts from the skin, indirectly supports the presence of this signaling pathway in dermal tissue.^75^ Thus, the “Piezo1-Akt-GSK3β-cyclin D1” pathway serves as a fundamental mechanism for the mechanically induced proliferation of dermal fibroblasts, backed by a robust theoretical basis.

Moreover, pharmacological investigations have reinforced the importance of this signaling pathway. The PI3K inhibitor LY294002 is capable of entirely inhibiting the anti-apoptotic and pro-proliferative actions induced by the Piezo1 agonist Yoda1,^93^^,^^98^ highlighting that PI3K/Akt is a crucial signaling hub downstream of Piezo1. In conclusion, Piezo1 functions as a primary mechanosensor, merging mechanical stimuli with cellular decisions regarding survival and proliferation via the PI3K/Akt pathway, which plays a vital role in the physiological process of skin wound healing.

**Figure 3:**
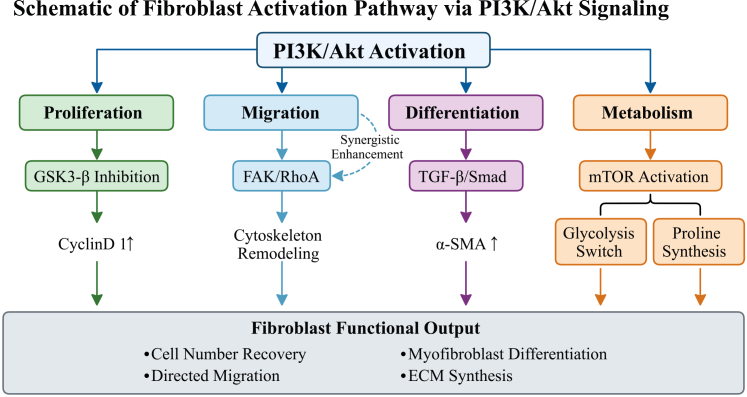
PI3K/Akt signaling hub orchestrates four major fibroblast functions Schematic overview of PI3K/Akt-mediated regulation of fibroblast activation and functional outcomes during wound healing. Proliferation: the activation of PI3K/Akt promotes fibroblast proliferation through various downstream mechanisms. The phosphorylation and inhibition of GSK3-β by Akt stabilize Cyclin D1, facilitating G1/S cell cycle progression and enabling recovery of cell numbers in the wound bed. Migration: PI3K/Akt signaling coordinates directed fibroblast migration through the activation of the FAK/RhoA pathway. This activation results in cytoskeletal reorganization and polarized cell movement toward the wound center, which is essential for wound closure. Metabolism: Akt activates mTOR signaling, inducing a metabolic switch that enhances glycolysis, akin to the Warburg effect. This metabolic reprogramming increases proline synthesis, providing essential substrates for collagen production and supporting the high biosynthetic demands of activated fibroblasts. Differentiation: PI3K/Akt acts synergistically with the TGF-β/Smad pathway to promote myofibroblast differentiation. This cooperation enhances the expression of α-smooth muscle actin (α-SMA), a hallmark of the myofibroblast phenotype, which is critical for wound contraction. Fibroblast functional output: the coordinated activation of these four pathways converges on the ultimate functional outcomes: ECM synthesis and tissue remodeling. Together, these processes ensure proper wound repair through balanced proliferation, migration, metabolic adaptation, and differentiation of fibroblasts.

In order to avoid the risk of confusing “PI3K/Akt is important in a broad sense” with “Piezo1 → PI3K/Akt is the main mechanical pathway,” we now clearly distinguish three levels of evidence for each functional output (proliferation/migration/metabolism/differentiation): (a) PI3K/Akt is required in wound fibroblasts; (b) Piezo1 is required; and (c) Piezo1 acts through PI3K/Akt (epistasis logic) (Table 3 and Figure 3).

**Table 3:** Evidence hierarchy for the Piezo1-PI3K/Akt axis in wound fibroblast function

Functional output	Tier 1: PI3K/Akt required in wound fibroblasts?	Tier 2: Piezo1 required?	Tier 3: Piezo1 acts through PI3K/Akt (epistasis)?
Proliferation	**Yes.** LY294002 blocks Piezo1-induced anti-apoptotic and pro-proliferative effects in fibroblast models (Zeng 2022; Zhan 2024). PI3K/Akt-Cyclin D1 axis confirmed across fibroblast types (Liu 2025).	**Yes.** Piezo1 siRNA inhibits stretch-induced proliferation in CD74^+^ keloid fibroblasts (Zhang 2024). GsMTx4 reduces fibroblast proliferation in HTS models (He 2021).	**Partial.** Zhang et al. (2024) showed Piezo1 knockdown reduces Akt phosphorylation and proliferation (Zhang 2024), but formal epistasis (Piezo1 activation + PI3K inhibitor rescue) has not been performed.
Migration	**Yes.** LY294002 inhibits dermal fibroblast migration *in vitro* and wound contraction *in vivo* (Li 2016).	**Yes (context-dependent).** Piezo1 knockout increases pseudopodia but reduces migration directionality in MEFs (Yang 2022). In keratinocytes, Piezo1 deletion enhances migration (Chen 2024)—opposite effect.	**Not directly tested.** The Piezo1 → Ca^2+^ → FAK → PI3K chain is supported step-by-step (Yao 2022; Chen 2023; Li 2016), but no single study has applied Piezo1 activation + PI3K/FAK inhibitor to demonstrate epistasis in fibroblast migration.
Metabolic reprogramming	**Partial.** Akt/mTOR activation broadly enhances glycolysis; Xue et al. (2025) linked Piezo1-Akt to Glut1/glycolytic enzyme upregulation in dermal cells (Xue 2025). Direct pharmacological block of PI3K on metabolic reprogramming has not been formally tested in wound fibroblasts.	**Yes.** Piezo1 deletion reduced glycolytic gene expression in skin (Xue 2025).	**Partial.** Xue et al. (2025) demonstrated Piezo1-Akt dependency (Xue 2025), but whether PI3K inhibition fully abolishes Piezo1-driven metabolic changes in fibroblasts specifically requires confirmation.
Myofibroblast differentiation	**Yes.** LY294002 blocks TGF-β-induced α-SMA expression and collagen gel contraction in dermal fibroblasts (Li 2016; Conte 2011). PI3K/Akt synergizes with Smad3 (Luo 2017).	**Yes.** Piezo1 knockout in Periostin^+^ myofibroblasts reduces pro-fibrotic gene expression (lung model) (Xu 2025). In skin, GsMTx4 prevents HTS formation (He 2021).	**Not directly tested.** The complete chain Piezo1 → Ca^2+^ → PI3K/Akt → Smad3 enhancement → α-SMA has not been demonstrated using epistasis logic in a single dermal fibroblast experiment. The strongest skin-specific evidence is the ILK-PI3K/Akt–myofibroblast link (Li 2016), with Piezo1 involvement inferred but not formally connected.

Note: epistasis validation requires rescue experiments combining Piezo1 agonist activation with PI3K/Akt inhibitor treatment in the same fibroblast system. HTS, hypertrophic scar; MEF, mouse embryonic fibroblast; α-SMA, alpha smooth muscle actin; GsMTx4, mechanosensitive Piezo1 channel blocker. Citation corrections applied: CD74^+^ keloid fibroblast data referenced as Zhang et al., 2024^75^; HTS GsMTx4 data as He et al., 2021^99^; MEF curvature data as Yang et al., 2022^100^; keratinocyte migration data as Chen et al., 2024^101^; lung Piezo1 knockout as Xu et al., 2025^102^; metabolic reprogramming as Xue et al., 2025^103^; PI3K/Akt-Smad3 synergy as Luo, 2017^104^ and Li et al., 2016.^105^

## Cytoskeletal remodeling and migration

A crucial aspect of the healing process for wounds involves the guided movement of fibroblasts toward the center of the injury.^106^ This migration is influenced by the active restructuring of the cytoskeleton and the formation of cell polarity. The signaling pathway involving Piezo1 and PI3K/Akt is significant in facilitating fibroblast movement by modulating the dynamics of the cytoskeleton.

### Evidence for the existence of the Piezo1/FAK/Akt axis

Zhang and colleagues validated the presence of the Piezo1/FAK/Akt signaling pathway in glioblastoma models.^107^ It is important to recognize that signaling pathways in cancer cells frequently exhibit abnormal activation, and their underlying mechanisms may not be relevant to the processes of normal tissue repair. Nonetheless, the subsequent evidence bolsters the idea that this signaling pathway plays a role in the migration of dermal fibroblasts:

To begin with, focal adhesion kinase (FAK) serves as a crucial integrin signaling center and a primary regulator of cell movement, playing a vital role in the mechanotransduction of fibroblasts. As previously noted, Yao et al. demonstrated that Piezo1 can associate with established focal adhesions in response to mechanical forces, with its linker domain (amino acids 1,418–1,656) being essential for these focal adhesion interactions.^67^ Additionally, research by Chen et al. revealed that the calcium influx mediated by Piezo1 can significantly boost the phosphorylation levels of FAK at Y397.^72^ It has been shown that FAK phosphorylation can directly trigger the activation of the PI3K/Akt signaling pathway by recruiting the p85 subunit of PI3K.^73^^,^^74^ Thus, the molecular framework for the signaling pathway “Piezo1 → Ca^2+^ → FAK → PI3K/Akt” in dermal fibroblasts has been partially characterized, though the complete sequence remains to be validated as a continuous chain within a single experimental model.

### Direct evidence in cutaneous wound healing

In the realm of skin wound healing, significant validation was provided by Li et al. Their research demonstrated that the PI3K/Akt inhibitor LY294002 effectively hinders the migration of dermal fibroblasts and slows down the processes of wound re-epithelialization and contraction *in vivo*.^105^ This finding suggests that the PI3K/Akt pathway plays a crucial role in the movement of dermal fibroblasts. When considered alongside the previously discussed Piezo1-Ca^2+^-FAK-PI3K signaling pathway, it can be reasonably concluded that Piezo1 influences the migration behavior of dermal fibroblasts through this mechanism.

### Spatial localization mechanism of Piezo1 in cell migration

Importantly, the influence of Piezo1 on cell movement is determined not just by its ability to allow ion flow but is also limited by its unique localization on the cell membrane. Advanced imaging techniques have revealed that Piezo1 is concentrated at the tips of lamellipodia and filopodia at the forefront of migrating cells rather than being evenly spread. In this region, Piezo1 detects membrane tension and facilitates the creation of localized calcium microdomains, which in turn promotes actin polymerization and swift changes in the cytoskeleton.^108^

In their 2022 study, Yang et al. uncovered a distinctive mechanism of “curvature-dependent sorting” associated with Piezo1 in mouse embryonic fibroblasts. The Piezo1 trimer, characterized by its large dome-like shape, preferentially localizes to areas of the membrane that are either flat or concave, while it is naturally excluded from regions with high curvature, such as outward protrusions.^100^ This physical characteristic likely plays a role in preventing the random activation of Piezo1 at the tips of pseudopodia, which is crucial for maintaining the directional movement of cells. The absence of Piezo1 resulted in a notable increase in the formation of fibroblast pseudopodia, but a reduction in the directionality of migration.

Remarkably, the research revealed that the agonist Yoda1 can trigger structural alterations in Piezo1, causing it to become “flattened” and enabling its entry into filopodia. This suggests that the therapeutic action of Yoda1 in wound healing could operate through two mechanisms: stimulating the PI3K/Akt pathway via calcium influx to boost cell movement, and starting mechanosignal transduction at the tips of pseudopodia by modifying the subcellular positioning of Piezo1, which in turn transforms the exploratory behavior of fibroblasts.

### Cell type-specific considerations

The effect of Piezo1 on cell movement is specific to different cell types. Research by Chen et al. on skin wound healing revealed that in keratinocytes, Piezo1 accumulates at the back of the cell, which slows down migration by promoting local retraction. In contrast, the absence of Piezo1 led to an increase in both the speed and directness of keratinocyte migration.^101^

This observation highlights that identical signaling pathways can yield vastly different or even contradictory functional results across various cell types. In keratinocytes, the Piezo1-Ca^2+^ signaling seems to mainly function as a “brake” by directly influencing the contractile force of myosin II. Conversely, in fibroblasts, the Piezo1-PI3K/Akt pathway generally facilitates migration and invasion, likely due to the role of PI3K/Akt in enhancing the formation and stabilization of pseudopodia. These distinctions between cell types may indicate the unique functional contributions each cell type makes during wound healing: keratinocytes need tightly controlled “collective migration” for effective re-epithelialization, while fibroblasts must possess a robust invasive ability to navigate through the provisional matrix and remodel the tissue architecture.

## Fibroblast activation and matrix synthesis

Activated fibroblasts, referred to as myofibroblasts,^109^ play a crucial role in the early stages of tissue repair by kick-starting the reconstruction of the ECM through the deposition of fibrous collagen.^110^ This process establishes an initial framework that compensates for the absent matrix and aids in restoring the structural integrity of the tissue. As this initially pliable matrix undergoes remodeling and becomes crosslinked into scar tissue capable of withstanding greater mechanical stress, the increased stiffness of the surrounding microenvironment prompts a phenotypic shift in fibroblasts. This transformation leads to the formation of contractile structures, known as stress fibers, composed of actin and myosin within the cells, enhancing their traction and contractile abilities—hallmarks of myofibroblasts.^111^

### Cooperative action of TGF-Β/SMAD pathway and mechanical force

TGF-β plays a crucial role in the activation of fibroblasts and their conversion into myofibroblasts, facilitating epithelial-mesenchymal transition, fibroblast activation, and ECM accumulation via both SMAD-dependent and alternative pathways. Additionally, it stimulates p38 MAPK, JNK, and PI3K/Akt, which enhances cytoskeletal changes and cell movement.^112^

In a groundbreaking study, Xu et al. revealed significant findings related to a pulmonary fibrosis model. Their research demonstrated that the differentiation of fibroblasts into myofibroblasts, driven by TGF-β, led to a notable increase in cell membrane tension. Crucially, the targeted deletion of Piezo1 in myofibroblasts that express Periostin resulted in a marked decrease in the levels of pro-fibrotic genes, including Postn, Fn1, Col1a1, Col3a1, Acta2, and Tgfb1. Additionally, this knockout effectively suppressed the activation of the TGF-β-Smad2/3 pro-fibrotic pathway.^102^ This research established, for the first time *in vivo*, a direct regulatory link between “Piezo1 and TGF-β/Smad”, suggesting that Piezo1 functions not just as a mechanoreceptor but also as a key upstream regulator of the TGF-β signaling cascade.

At the molecular scale, there exists a complex interplay between the PI3K/Akt and Smad signaling pathways, which offers insight into how Piezo1-Ca^2+^ signals contribute to the differentiation of myofibroblasts. In a comprehensive review by Luo, this network of signal interactions is thoroughly discussed: TGF-β can stimulate Akt via pathways involving integrin-linked kinase (ILK) and other mediators; once activated, Akt boosts the transcriptional function of Smad3 by phosphorylating specific regions in the Smad3 linker, rather than at the C terminus, leading to the upregulation of target genes like Collagen I. Importantly, merely activating the PI3K-Akt pathway does not suffice to elevate gene expression; its role in enhancing Smad3 activity is crucial for achieving optimal Smad3 activation.^104^ This observation underscores the role of PI3K/Akt as a “signal amplifier” within the TGF-β/Smad signaling cascade.

In their 2011 study, Conte and colleagues conducted direct functional validation using human lung fibroblasts. When these isolated fibroblasts were exposed to TGF-β at a concentration of 10 ng/mL for 48 h, they showed increased rates of proliferation and transitioned into myofibroblast phenotypes, which were marked by the expression of α-SMA and the production of collagen.^45^ This transformation was associated with a notable rise in pAkt levels. Importantly, the use of the pan-PI3K inhibitor LY294002 during treatment completely inhibited the TGF-β-induced enhancements in cell growth, α-SMA expression, and collagen accumulation. This finding strongly indicates that the activation of the PI3K/Akt pathway is essential for the differentiation of myofibroblasts induced by TGF-β, rather than being a mere byproduct of the process.

The study conducted by Guo and colleagues expanded on this knowledge by demonstrating that the transformation of normal human lung fibroblasts into myofibroblasts, triggered by TGF-β1, relies on the activity of HDAC4 and the phosphorylation of Akt. The use of TSA, an HDAC inhibitor, led to a decrease in α-SMA expression, which was linked to lower levels of Akt phosphorylation. Additionally, the application of pharmacological inhibitors targeting Akt effectively hindered the TGF-β1-induced α-SMA expression in a dose-dependent manner, further emphasizing the critical role of Akt phosphorylation in regulating α-SMA levels.^113^

The mechanistic investigations mentioned above largely originate from models involving lung fibroblasts. In the realm of skin wound healing, Li et al. were pioneers in systematically establishing the pivotal function of the ILK-PI3K/Akt pathway in the contraction of skin wounds. Their research revealed that ILK exhibited variable expression at various stages of wound healing in rat skin, with its levels showing a positive correlation to the rate of wound contraction. *In vivo* studies demonstrated that the ILK inhibitor QLT0267 and the PI3K/Akt inhibitor LY294002 both significantly impeded wound contraction and re-epithelialization, thus prolonging the healing process.^105^ Additionally, *in vitro* experiments validated that these inhibitors could reduce contraction in fibroblast-populated collagen gels, hinder fibroblast migration, and prevent TGF-β1-induced α-SMA expression in normal dermal fibroblasts. Furthermore, the application of ILK siRNA to directly inhibit ILK expression also led to a notable reduction in both dermal fibroblast migration and α-SMA expression.^105^ Collectively, these findings robustly support the notion that the ILK-PI3K/Akt signaling pathway plays a crucial role in mediating skin wound contraction by influencing fibroblast migration and their differentiation into myofibroblasts.

Based on the previously discussed evidence regarding Piezo1’s function in dermal fibroblasts, a comprehensive signaling framework can be outlined: mechanical stress → activation of Piezo1 → influx of Ca^2+^ → (via CaM/CaMKII or integrin-FAK pathways) → activation of PI3K/Akt → increased TGF-β/Smad signaling → transcription of α-SMA → differentiation into myofibroblasts. This proposed sequence suggests a mechanism by which fibroblasts may accurately detect mechanical signals in the wound environment and potentially undergo phenotypic changes to facilitate tissue healing.

### Molecular regulation of extracellular matrix synthesis

The efficiency of ECM production is directly influenced by the Piezo1-PI3K/Akt signaling pathway, which modulates the mechanisms of protein translation. In their study, Byun et al. explored this “Piezo1-Akt-mTOR” synthesis pathway within a skin regeneration model using PLLA as a biomaterial.

Research indicated that the mechanical stimulation from PLLA microspheres stimulated Piezo1 channels on the membranes of fibroblasts, resulting in an influx of Ca^2+^ and a notable increase in Akt phosphorylation. The activated Akt subsequently triggered the mTORC1 complex, which in turn elevated the expression of crucial downstream translation regulatory factors, p-S6K1 and p-4EBP1. This signaling pathway was reported to initiate the intracellular protein translation process, with notable enhancement in the production and release of type I and type III collagen observed in this biomaterial model.^114^

### Metabolic reprogramming and energy supply

Beyond the activation of fibroblasts, the regulation of metabolism is crucial for the healing process of wounds. Changes in the metabolic pathways of fibroblasts, especially shifts in fatty acid oxidation and glycolysis, have a direct impact on the production and breakdown of ECM elements.^115^

In their 2025 study, Xue and colleagues demonstrated that the activation of Akt by Piezo1 is crucial for substantial metabolic changes. When subjected to mechanical stress, Piezo1 enhanced the expression of the glucose transporter Glut1 (Slc2a1) and essential glycolytic enzymes, including Aldoa and Ldha, in dermal cells via calcium signaling pathways. This metabolic shift, orchestrated by the Piezo1-Akt pathway, supplies vital carbon sources and energy necessary for tissue repair.^103^

In addition to energy needs, the production of collagen depends on certain amino acids, particularly proline. Research by He et al. highlighted the crucial regulatory function of Piezo1 in the “arginine-proline metabolic pathway” within a model of skin fibrosis.

Research indicated that treatment with a high-stiffness matrix or the Piezo1 agonist Yoda1 notably increased the levels of essential metabolic enzymes such as Arg1 (arginase 1), OAT (ornithine aminotransferase), and PYCR1 in fibroblasts. This process facilitated the transformation of L-arginine into L-proline, resulting in an increase in intracellular proline reserves, which in turn supplied adequate material for collagen production.^116^

### Mechanical imbalance and pathological outcomes

Successful healing of wounds depends on the swift cessation of mechanical signals once tissue repair is finished. In contrast, faulty mechanotransduction can lead to detrimental changes in tissue structure. Recent studies indicate that the Piezo1-PI3K/Akt signaling pathway experiences dysregulation in both directions during abnormal wound healing: overactivation can lead to fibroproliferative conditions like hypertrophic scars (HTSs) and keloids, while inadequate mechanotransduction is linked to the halted healing seen in chronic wounds. Exploring these opposing pathological conditions through the perspective of Piezo1-driven mechanotransduction offers a cohesive understanding of the various clinical outcomes associated with improper wound healing.

#### Excessive mechanotransduction: Positive feedback loops in fibrotic scarring

A study conducted by Li et al. uncovered a crucial pathological mechanism linked to the rigidity of the ECM. This stiff ECM not only activates Piezo1 channels but also reduces the function of its negative regulators through the Integrin β1/miR-369-3p pathway, leading to an overproduction of Piezo1 protein. This process, termed “stiffness-driven receptor upregulation,” establishes a dangerous positive feedback loop: as matrix stiffness increases, Piezo1 expression rises, which further enhances Piezo1 signaling and triggers the release of downstream effectors (such as VEGF or collagen) that promote additional matrix stiffening.^117^

He et al. expanded on this idea by clarifying a distinct molecular pathway that drives the positive feedback loop in skin fibrosis. Their research revealed a significant increase in Piezo1 expression in human HTSs and keloids, particularly in myofibroblasts. The authors pinpointed the Piezo1-Wnt2/Wnt11-CCL24 signaling pathway as a crucial factor in mechanically induced skin fibrosis. Activation of Piezo1 on rigid surfaces prompted dermal fibroblasts to release C-C motif chemokine ligand 24 (CCL24, also referred to as eotaxin-2), a strong pro-fibrotic cytokine, via Wnt2 (canonical β-catenin pathway) and Wnt11 (non-canonical JNK pathway) pathways. Notably, CCL24 could activate dermal fibroblasts in an autocrine or paracrine fashion, and the resulting tissue stiffening further elevated Piezo1 levels, creating a self-reinforcing cycle. The use of adeno-associated virus (AAV) to knock down Piezo1 significantly reduced skin fibrosis and stiffness in a mouse model,^118^ offering concrete evidence that targeting Piezo1 can disrupt this harmful feedback loop in dermal tissue.

He et al. provided direct evidence for the involvement of Piezo1 in the development of HTSs, revealing its elevated expression in the myofibroblasts of HTS tissues from both humans and rats. Their *in vitro* studies indicated that cyclic mechanical stretch significantly enhanced Piezo1 levels and calcium influx dependent on Piezo1 in human dermal fibroblasts, which in turn facilitated cell proliferation, migration, differentiation, and collagen synthesis. Importantly, the use of the Piezo1-inhibiting peptide GsMTx4 *in vivo* successfully shielded rats from HTS formation triggered by stretching,^99^ highlighting the potential of Piezo1 inhibition as an effective therapeutic approach to prevent scarring caused by mechanical forces.

Recent studies on the keloid microenvironment have uncovered a new mechanotransduction pathway involving Piezo1. Research by Liao et al. indicated that Piezo1 facilitates the transfer of mechanical signals, which enhances the nuclear translocation of YAP via calcium influx. This process activates a signaling axis comprising Piezo1, calcium, and YAP, which promotes the synthesis of matrix components that are both proadhesive and profibrotic.^119^ This discovery adds to the previously discussed PI3K/Akt pathways and implies that Piezo1 may serve as a key intersection for various pro-fibrotic signaling pathways, including PI3K/Akt and Hippo/YAP, within pathological scar tissue.

In a study conducted by Zhang et al., researchers discovered a unique group of CD74^+^ fibroblasts within keloids that exhibit a heightened sensitivity to mechanical stretching. Their research revealed that mechanical stimuli triggered the activation of Piezo1 channels located in the cell membrane, resulting in an influx of calcium ions and the activation of various downstream signaling pathways, such as Akt phosphorylation and the ERK pathway.^75^ This Piezo1-Akt signaling mechanism was shown to directly promote fibroblast proliferation and the secretion of pro-angiogenic factors, contributing to the abnormal formation of scar tissue. Furthermore, the investigation indicated that a reduction in Piezo1 expression led to a significant decrease in stretch-induced Akt phosphorylation and fibroblast growth, suggesting that targeting this signaling pathway may offer a viable strategy for addressing excessive wound healing.

#### Insufficient mechanotransduction: Piezo1-PI3K/Akt dysfunction in chronic non-healing wounds

Unlike the excessive activation seen in fibrotic scars, persistent non-healing wounds such as diabetic foot ulcers, pressure ulcers, and chronic venous ulcers may indicate a lack of or irregular mechanotransduction. While there is currently insufficient direct evidence connecting Piezo1 dysfunction to the reduced activity of dermal fibroblasts in these chronic wounds, various research findings collectively bolster this theory.

The microenvironment of diabetic wounds shows a notable disruption in the PI3K/Akt signaling pathway. Studies indicate that fibroblasts exposed to high glucose levels demonstrate significant dysfunction in the PI3K/Akt and the subsequent mTOR/GSK3β pathways when compared to those in normal glucose conditions, leading to diminished abilities in proliferation and migration.^120^ Furthermore, research by Bitar and Al-Mulla revealed that fibroblasts exhibiting traits of type 2 diabetes have a marked reduction in IGF1-induced activation of IRS1, along with impaired phosphorylation of PI3K and Akt. This impairment is linked to ROS-induced activation of JNK and the inhibitory serine phosphorylation of IRS1.^121^ These observations imply that the downstream signaling pathway of Piezo1 is already weakened in diabetic fibroblasts, which could significantly reduce their responsiveness to mechanical signals during the healing process.

#### Aberrant mechanical cell-cell interactions in pathological remodeling

Changes in mechanotransduction are apparent not only in the physical changes of ECM stiffness but also in the unusual reorganization of cellular interaction patterns. Research by Ezzo et al. revealed that the mechanical interaction between pro-fibrotic macrophages and fibroblasts can swiftly activate calcium signaling through Piezo1, resulting in fibrotic responses. More specifically, the binding of αVβ3 integrins on macrophages to the fibroblast surface mechanically stimulates Piezo1, leading to intracellular Ca^2+^ fluctuations that promote myofibroblast differentiation and ECM synthesis.^122^ This process is particularly important as it illustrates that Piezo1-mediated mechanotransduction goes beyond cell-matrix interactions, incorporating direct mechanical communication between cells, thereby adding complexity to the mechanisms governing wound healing.

This finding carries significant implications for our comprehension of chronic wounds and abnormal scar formation. In the case of chronic wounds, the ongoing inflammatory microenvironment is dominated by a high presence of proinflammatory macrophages.^123^ The continuous mechanical interactions between these activated macrophages and local fibroblasts may create a detrimental cycle of inflammatory signaling mediated by Piezo1,^124^ On the other hand, in conditions like keloids and HTSs, the ongoing mechanical pressure and release of proinflammatory cytokines via Piezo1 can result in the prolonged activation of the PI3K/Akt signaling pathway and the enduring myofibroblast state, hindering the normal progression of the wound healing process.

### Opposing Piezo1 modulation requirements in chronic wounds versus fibrotic scars

The preceding discussion reveals a fundamental therapeutic dilemma: chronic non-healing wounds appear to be characterized by insufficient Piezo1-PI3K/Akt signaling, suggesting a need for pathway activation, whereas HTSs and keloids are driven by excessive mechanotransduction through the same axis, warranting pathway inhibition. This bidirectional dysregulation creates what we term a “therapeutic paradox”—the same molecular target requires opposite interventions depending on the clinical context.

Several strategies may help resolve this paradox. First, temporal phase-specific modulation could exploit the distinct healing stages as therapeutic windows. During the early inflammatory and proliferative phases, when fibroblast recruitment and ECM deposition are essential, transient Piezo1 activation (e.g., via Yoda1 or mechanotherapeutic devices) may restore the diminished mechanosensitivity observed in diabetic or aged wounds. Notably, recent work has demonstrated that Piezo1-mediated mechanotransduction in aging wounds promotes ferroptosis in endothelial cells through the CaMKII/ATF3/SLC7A11 axis, and that pharmacological inhibition of Piezo1 protects aged tissues from ferroptotic damage.^125^ This finding further underscores the need for time-restricted Piezo1 activation: even in chronic wounds, prolonged or unregulated Piezo1 stimulation could paradoxically impair healing by triggering ferroptosis in senescent cells. Conversely, during the remodeling phase, when ECM crosslinking and tissue stiffening progressively increase, Piezo1 antagonism (e.g., GsMTx4) may be administered to interrupt the stiffness-driven positive feedback loop that promotes pathological scarring. The clinical experience with scar compression therapy, which applies sustained mechanical offloading during remodeling, provides indirect support for this phase-specific concept.^123^

Regarding cell-type specificity, Piezo1 exerts opposing functional effects across cell types within the same wound: it facilitates fibroblast migration but inhibits keratinocyte migration (section 4.2.4), and its activation in pro-fibrotic macrophages triggers fibroblast differentiation through direct cell-cell contact.^122^ In immune cells, Piezo1 regulates ILC2 effector function through mTOR-dependent translational control, with conditional Piezo1 deletion attenuating lung fibrosis.^124^ These divergent roles necessitate cell-selective approaches, such as fibroblast-specific AAV vectors (as demonstrated by He et al. in 2024 for dermal Piezo1 knockdown^118^) or ligand-functionalized nanoparticle delivery systems.

Finally, it should be emphasized that the therapeutic paradox extends beyond Piezo1 to the broader PI3K/Akt signaling node. Given that multiple upstream inputs converge on this pathway, interventions targeting downstream effectors (e.g., mTOR for metabolic reprogramming, GSK3β for proliferation) rather than Piezo1 itself may offer finer therapeutic control with reduced risk of disrupting the diverse physiological roles of the channel. Future studies should prioritize *in vivo* wound models that incorporate temporal and spatial monitoring of Piezo1-PI3K/Akt activity across healing phases to empirically define the optimal therapeutic windows and delivery parameters.

## Integration of the Piezo1-PI3K/Akt axis with parallel mechanotransduction pathways

### Crosstalk with YAP/TAZ pathway

The Hippo-YAP/TAZ pathway is a well-established mechanotransduction route that translates matrix stiffness into transcriptional programs governing fibroblast activation.^13^^,^^126^ Akt can directly phosphorylate and inhibit LATS1/2, promoting YAP nuclear translocation. In the Piezo1 context, Ca^2+^ influx may simultaneously activate both PI3K/Akt and YAP/TAZ pathways. Recent work has identified novel mechanotransduction pathways in stem cell regulation^127^ and integrated mechanosignaling networks^128^ that further underscore these interconnections. Future studies employing dual-reporter systems and time-resolved phosphoproteomics will be essential.

### Crosstalk with RHO/ROCK pathway

CADM1-prolonged Piezo1 opening may sustain FAK/RhoA/ROCK cascade, creating a feedforward loop: Piezo1 → Ca^2+^ → RhoA/ROCK → increased membrane tension → Piezo1 reactivation. ROCK also modulates PI3K/Akt by phosphorylating PTEN and altering focal adhesion distribution. A recent study reveals new dimensions of this crosstalk.^129^ Conversely, PI3K/Akt feeds back through Akt-mediated phosphorylation of Rac1/Cdc42 regulators, balancing protrusion and retraction during migration.

### Crosstalk with MAPK/ERK pathway

Zhang et al. showed that mechanical stretching of CD74^+^ keloid fibroblasts activated both Akt and ERK pathways,^75^ suggesting parallel activation downstream of Piezo1. As before, P2Y2 can also activate ERK through Gq-dependent and RGD-integrin routes.^85^^,^^90^^,^^91^

## Future directions and clinical application prospects of Piezo1

Piezo1 demonstrates varying effects that can be tissue-specific and sometimes contradictory; however, certain mechanistic trends are reliably identified. Evidence to date suggests that, acting as a mechanosensor, it may detect alterations in tissue rigidity and the composition of the ECM, facilitating the influx of Ca^2+^, which has been proposed to serve as a key initiator for subsequent signaling pathways in a context-dependent manner. Important pathways involved include MAPK, TGF-β, YAP/TAZ, PI3K/Akt, and fibroblast activation, which can either promote wound healing or contribute to abnormal scarring, depending on the cellular environment. Although some mechanisms, particularly those related to PI3K/Akt, are not yet completely understood, these common signaling characteristics lay the groundwork for comprehending its dual role in the wound healing process.^130^

Piezo1 serves a crucial function as a transducer of mechanical forces, making it a highly promising yet under-researched target for therapy. Although interest in Piezo1 as a potential therapeutic avenue is increasing, the development of drugs remains fraught with obstacles. Recent investigations have repositioned Piezo1 from a passive mechanosensor to a versatile therapeutic switch across diverse pathologies. In the context of cancer immunotherapy, particularly for non-small cell lung cancer liver metastasis, pharmacological activation of Piezo1 has emerged as a promising strategy to overcome matrix stiffness-mediated immunosuppression. By modulating the PD-L1/CXCL10 axis, this approach effectively sensitizes stiff tumors to checkpoint blockade.^126^ The principle of harnessing mechanical signaling extends to the maintenance of tissue integrity, where Piezo1 agonists have been shown to reinforce intercellular junctions against destructive stress in Hailey-Hailey disease.^4^ However, in wound repair, Piezo1 activity requires precise regulation to balance efficient healing with the prevention of pathological scarring.^123^ Furthermore, targeting the Piezo1-mediated mechanotransduction-translation axis in immune cells, such as ILC2s, presents a novel avenue to mitigate respiratory inflammation.^124^ Collectively, these findings underscore the potential of Piezo1 modulators to treat a spectrum of diseases driven by aberrant mechanosensing, ranging from solid tumors to genetic dermatoses. Existing modulators like Yoda1 and GsMTx4 are limited by their lack of selectivity, potential off-target effects, and unsuitability for systemic administration due to issues with stability and bioavailability.^131^^,^^132^^,^^133^ This highlights the urgent need for strategies that enable precise targeting of tissues. Additionally, the absence of clinically validated compounds continues to impede therapeutic investigations *in vivo*. Natural compounds such as paeoniflorin and artemisinin have demonstrated the ability to interact with the Piezo1 pore region, effectively inhibiting calcium influx triggered by mechanical forces or chemical agonists.^134^^,^^135^ Consequently, future studies should prioritize *in vivo* models that explore the spatial structure of ligand-channel interactions and enhance pharmacokinetics through targeted delivery methods. Similarly, in terms of wound care, we may be able to use Piezo1 modulators in combination with physical models (such as hyperbaric oxygen and strontium zinc silicate-based biomaterials)^136^^,^^137^ in the future, which may provide a synergistic approach to promote wound healing.

## Conclusion

This review comprehensively examines the molecular processes and functional roles of the Piezo1-Ca^2+^-PI3K/Akt signaling pathway in skin wound healing. Piezo1, a distinct MAC characterized by its intricate “blade-beam-pore” trimeric configuration, has been proposed to translate mechanical stress signals from the ECM into intracellular Ca^2+^ transient signals that appear to exhibit specific spatiotemporal patterns. Available evidence suggests that this calcium signaling may engage various parallel or sequential molecular pathways. These include the direct activation route via CaM/CaMK, the integrin-FAK cooperative mechanism, the calpain regulatory system, and the ATP-P2Y2 paracrine pathway. Together, these routes may converge on the PI3K/Akt signaling node. Once activated, the PI3K/Akt pathway has been implicated in orchestrating several key fibroblast functions. At the level of cell survival and proliferation, the Akt-GSK3β-Cyclin D1 cascade promotes cell cycle progression. Cytoskeletal remodeling mediated via FAK may further enhance directional migration toward the wound center. In addition, PI3K/Akt appears to act synergistically with the TGF-β/Smad pathway to facilitate myofibroblast differentiation. Finally, activation of the downstream mTOR pathway has been associated with collagen production and metabolic reprogramming, thereby supporting the biosynthetic demands of tissue repair.

Nonetheless, several critical challenges remain to be addressed. First, the precise molecular mechanism linking Piezo1-mediated Ca^2+^ signals to PI3K/Akt activation—particularly via the CaM-pY99-PI3K pathway—has yet to be directly validated in dermal fibroblasts. Second, the extent to which this signaling axis is dysregulated in chronic non-healing wounds and pathological scars remains unclear, and its therapeutic relevance awaits further *in vivo* investigation. Third, the effects of Piezo1 on cell migration appear to be highly cell type-specific: whereas Piezo1 activity tends to facilitate fibroblast migration, it has been reported to exert inhibitory effects in keratinocytes. Future therapeutic strategies should therefore account for such cell-type selectivity to avoid unintended consequences.

A thorough examination of the intricate regulatory framework surrounding the Piezo1-PI3K/Akt signaling pathway is essential for progressing in this area. This analysis is expected to offer fresh insights into the mechanobiological processes involved in skin wound healing and to lay a robust theoretical groundwork for creating innovative wound care approaches that leverage mechanotransduction. These may include the precise administration of Piezo1 modulators, the mechanical enhancement of biomaterial designs, among other strategies.

It is important to clarify our use of the term “signaling hub” in this review. For the purposes of this review, a mechanotransduction context-dependent hub is operationally defined as a signaling node satisfying one or more of the following criteria. Convergence refers to the capacity of multiple upstream mechanical inputs to funnel into a common pathway. Necessity implies that loss-of-function perturbation significantly attenuates mechanotransduction-dependent cellular responses. Sufficiency denotes that gain-of-function activation can at least partially recapitulate the effects of mechanical stimulation. Current evidence most strongly supports the convergence criterion for the Piezo1-PI3K/Akt axis in dermal fibroblasts, with necessity demonstrated primarily through pharmacological inhibition (e.g., LY294002). Sufficiency evidence remains limited. We therefore characterize Piezo1-PI3K/Akt as a “context-dependent convergent node” rather than asserting singular dominance over other mechanotransduction pathways (e.g., YAP/TAZ, Rho/ROCK, and MAPK).

## Acknowledgments

This study was supported by the Shanxi Provincial Basic Research Program (Free Exploration) (no. 2024030212211292) and the Shanxi Provincial Key Laboratory of Functional Proteins 3D (no. 2025SXKLFP006).

## Author contributions

Conceptualization, investigation, writing – original draft, writing – review and editing, and methodology, S.W.; conceptualization, writing – original draft, writing – review and editing, and visualization, Y.L.; writing – original draft, writing – review and editing, and formal analysis, Y.H.; conceptualization and writing – review and editing, S.L. and R.Z.; conceptualization, funding acquisition, project administration, resources, and writing – review and editing, Y.J.

## Declaration of interests

The authors declare that the research was conducted in the absence of any commercial or financial relationships that could be construed as a potential conflict of interest.
